# Risk factors for acquisition of carbapenem resistant Enterobacteriaceae in an acute tertiary care hospital in Singapore

**DOI:** 10.1186/s13756-015-0066-3

**Published:** 2015-06-23

**Authors:** Moi Lin Ling, Yong Ming Tee, Soong Geck Tan, Ismawati M. Amin, Kue Bien How, Kwee Yuen Tan, Lai Chee Lee

**Affiliations:** Infection Control, Singapore General Hospital, Outram Road, Singapore, 169608 Singapore

**Keywords:** Enterobacteriaceae, Carbapenem resistant, Risk factors, Epidemiology

## Abstract

**Background:**

Carbapenem resistant *Enterobacteriaceae* (CRE) is increasingly reported worldwide. A similar increase is seen in Singapore since identification of its first case in 2008. The aim of this study was to identify local risk factors for carriage of CRE in patients from an acute tertiary care hospital in Singapore.

**Method:**

A matched case-control study was conducted on inpatients treated from January 1, 2011 till December 31, 2013. Two hundred and three cases of CRE infection or colonization were matched with 203 controls. CRE types were identified by PCR. Statistical analysis of data including a multivariate logistic regression analysis was done using SPSS 21.0.

**Results:**

CREs were commonly seen in *Klebsiella pneumoniae* (42.2 %), *Escherichia coli* (24.3 %) and *Enterobacter cloacae* complex (17.2 %) in the 268 isolates. NDM-1 was the commonest CRE type seen (44.4 %), followed by KPC (39.9 %) whilst OXA-48 only constituted (7.8 %). Univariate analysis identified key risk factors associated with CRE as history of previous overseas hospitalization (OR: 33.667; 95 % CI: 4.539-259.700), admission to ICU (OR: 11.899; 95 % CI: 4.986-28.399) and HD/ICA (OR: 6.557; 95 % CI: 4.057-10.596); whilst a multivariate analysis revealed exposure to antibiotics penicillin (OR: 4.640; 95 % CI: 1.529-14.079] and glycopeptide (OR: 5.162; 95 % CI: 1.377-19.346) and presence of central line device (OR: 3.117; 95 % CI: 1.167-8.330) as significant independent predictors.

**Conclusions:**

The identification of risk factors amongst our local population helped to refine the criteria used for target active surveillance screening for CRE amongst inpatients at time of hospital admission.

## Introduction

Carbapenem resistant *Enterobacteriaceae* (CRE) is increasingly reported worldwide with increasing awareness of the global problem and improved methods of laboratory identification. Following the report of the emergence of the New Delhi metallo-β-lactamase-1 (NDM-1) in India, Pakistan, and the United Kingdom in 2010, there has also been much worldwide interest and concern of its increased epidemiology and potential impact on patient care. In Asia, *Klebsiella pneumoniae* Carbapenemase (KPC) was first detected in China in 2004 [1], and subsequently in South Korea and Taiwan [2, 3]. In Singapore, KPC was first reported in 2012 [4] where two of the four patients had strains closely related to the Chinese strain but the other two patients were non-Chinese with no travel history. The authors suggested possible community dissemination of KPC.

Shortly after the first discovery of the first NDM-1 patient at the Singapore General Hospital (SGH) in 2008, we noticed a steady gradual rise in the number of CRE patients in the subsequent years [5, 6]. This has significant implication on care of some of our patients requiring intermediate long term care (ILTC) management as some ILTCs were reluctant to receive CRE patients due to limited isolation facilities. Healthcare associated infections associated with multidrug resistant Gram negative bacilli have been shown to incur higher financial costs; although a significant proportion of which are subsidized by public funding in the form of governmental subvention in Singapore [7]. Moreover, there is also the risk of potential nosocomial CRE outbreaks arising from environmental contamination or lapses in infection control practices [8–11]. In light of these challenges and the fact that there are limited therapeutic options for the management of patients with CRE infections, we conducted a case-control study with the main objectives of identifying risk factors for carriage of CRE in our patients and enhancing existing strategies in controlling the spread of CRE amongst inpatients at our hospital.

## Methods

### Study design and population

We conducted a matched case-control study to identify risk factors associated with the acquisition of CRE amongst inpatients treated at SGH, a 1700-bed tertiary acute care hospital in Singapore. Cases were adults over age 18 years, whom CRE were isolated from clinical cultures from any source between January 1, 2011 and December 31, 2013. For each CRE patient, one control was randomly selected from adult inpatients admitted within the study period matched for gender, age, without CRE isolates. Subjects with CRE isolated from multiple sites or on multiple dates were counted only once where information from first event was collected as a case.

### Data collection

Data were extracted from the patients’ medical records and from hospital computerized databases according to a pre-prepared questionnaire. Variables analyzed as possible predictors included demographics (age, gender, ethnic group, ward class), specific co-morbid conditions (cardiovascular, renal, diabetes mellitus, malignancy, transplantation, etc.), length of stay, history of admission from overseas hospital in the past one year, history of admission in the past one year, history of overseas travel in the past one year, admissions to intensive care unit (ICU), high dependency or intermediate care area (HD or ICA), invasive procedures, surgical procedures, presence of other multidrug resistant organisms (MDROs), invasive devices (within one month prior to CRE) and exposure (≥1 day(s)) to antimicrobials (imipenem, meropenem, ertapenem, doripenem, ciprofloxacin, vancomycin, cephalosporins, piperacillin-tazobacatam, metronidazole), and radiation therapy (deep x-ray therapy DXT) (within one month prior to CRE identification).

### Microbiological methods

Carbapenem susceptibility was determined using disk diffusion and interpreted in accordance to the 2009 Clinical and Laboratory Standards Institute guidelines as per hospital’s clinical microbiology laboratory protocol. Carbapenemase-producing CRE were then identified using the modified Hodge test and the Roscoe test. Their CRE types were confirmed by the National Public Health Laboratory using the polymerase chain reaction (PCR) method.

### Data analysis

The association of categorical variables with CRE patients was first examined using Χ^2^or Fisher exact test and Odds ratio (ORs) analysis with corresponding 95 % confidence intervals (CIs) computed. For continuous data, Student *t* tests and Mann-Whitney *U* tests were applied appropriately. A multivariate logistic regression analysis evaluated the independent contribution of the variables if *p* < 0.1, adjusted for total length of hospital stay. Statistical significance was considered when *p* was less than 0.05. Statistical analyses were performed using SPSS 21.0(SPSS, Chicago, IL, USA).

## Results

### Demographic data

Two hundred and three cases of CRE infection or colonization were identified between 1 January 2011–31 December 2013 and matched with 203 controls. The distributions of age, race, gender and ward classes are shown in Table 1.

**Table 1:** Demographic details of CRE patients and control patients

Demographic	CP-CRE patients	Control	Standards	X-value
	(*N* = 203)	(*N* = 203)		(*p*-value)
	n (%)	n (%)	n (%)	(case vs. standards)
Mean age ± SD (years)	64.3 ± 16.0	64.0 ± 15.7	-	-
(mean years 95 % confidence interval)	(62.1–66.5)	(61.9–66.2)		
Gender				
Male	113 (55.7 %)	113 (55.7 %)	1,891,500 (49.2 %)^[a]^	3.331
Female	90 (44.3 %)	90 (44.3 %)	1,953,200 (50.8 %)^[a]^	(0.068)
Paying class				
(based on collection ward)				
Class A	16 (7.9 %)	26 (12.8 %)	5696 (7.2 %)^[b]^	
Class B1/B1+	20 (9.9 %)	15 (7.4 %)	9050 (11.5 %)^[b]^	9.086
Class B2	86 (42.4 %)	77 (37.9 %)	32,048 (40.8 %)^[b]^	(0.028)*
Class C	81 (39.9 %)	85 (41.9 %)	24,028 (30.6 %)^[b]^	
Ethnic group				
Chinese	134 (66.0 %)	133 (72.7 %)	2,853,800 (74.2 %)^[a]^	
Malay	29 (14.3 %)	32 (17.5 %)	512,800 (13.3 %)^[a]^	5.437
Indian	16 (7.9 %)	16 (8.7 %)	351,700 (9.1 %)^[a]^	(0.142)
Others (Singaporean)	1 (0.5 %)	2 (1.1 %)	126,500 (3.3 %)^[a]^	
Others: Bangladeshi	7 (3.4 %)	1 (0.5 %)	-	-
Others: Burmese	1 (0.5 %)	1 (0.5 %)	-	-
Others: Chinese	1 (0.5 %)	4 (1.9 %)	-	-
Others: Indian	2 (1.0 %)	2 (1.0 %)	-	-
Others: Indonesian	4 (1.9 %)	4 (1.9 %)	-	-
Others: Malaysian	2 (1.0 %)	0 (0 %)		
Others: Oman	1 (0.5 %)	0 (0 %)		
Others: Pakistani	2 (1.0 %)	0 (0 %)		
Others: Qatar	0 (0 %)	1 (0.5 %)	-	-
Others: Thai	0 (0 %)	1 (0.5 %)	-	-
Others: UAE	0 (0 %)	2 (1.0 %)		
Others: Vietnamese	3 (1.5 %)	3 (1.5 %)	-	-

*p < 0.05 (Significance of association; 2-tailed)

^a^Department of Statistics Singapore, 2013, *Population Trends 2013*

^b^SGH Bulletin Beds in Service by Ward and Bed Class (2013)

The common CRE isolated from 203 patients were *Klebsiella pneumoniae* (42.2 %), *Escherichia coli* (24.3 %) and *Enterobacter cloacae* complex (17.2 %). The cases were non-gender bias in the 32–96 years old age group with distribution as Chinese (66.0 %), Malay (14.3 %), Indian (7.9 %) and others (11.8 %). Average length of stay before the patient was identified with CRE was 21.1 ± 40.5 days.

#### Isolation sites and types of CRE

**Fig. 1:**
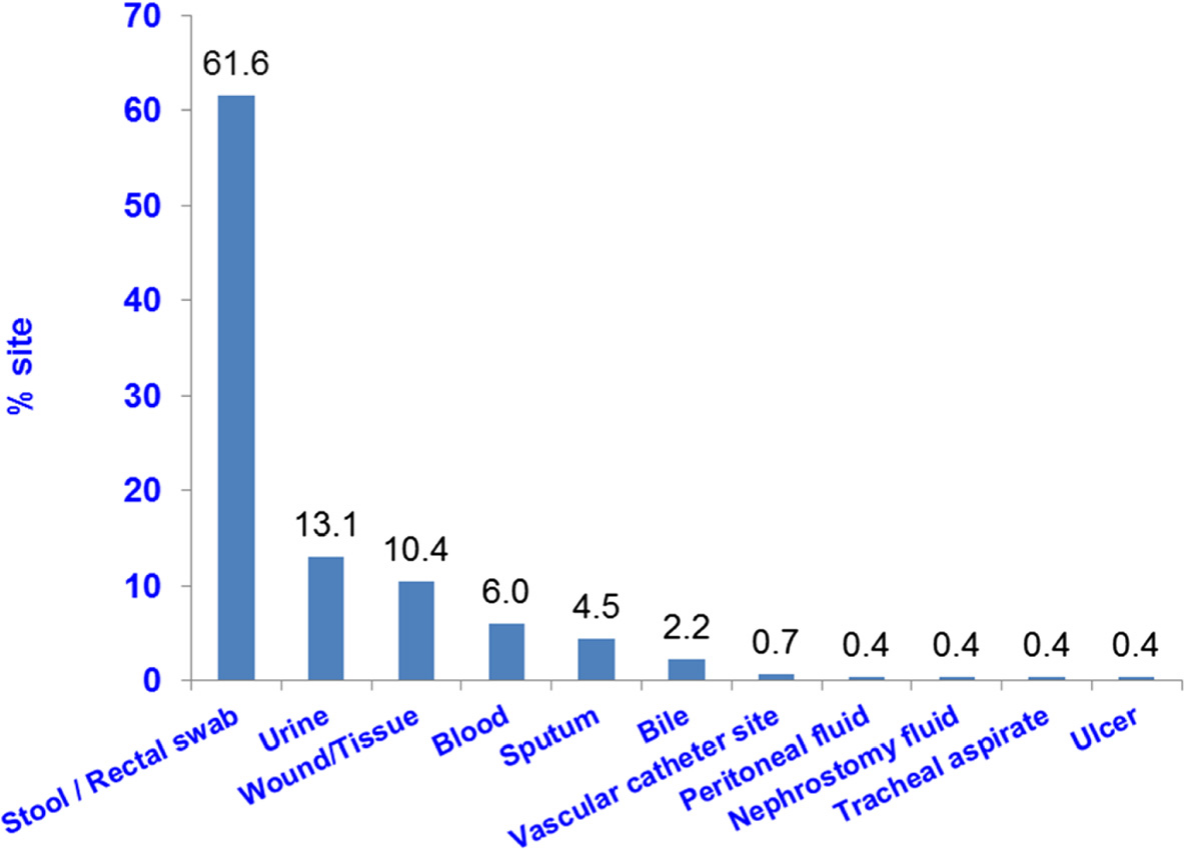
Distribution of CRE by isolation sites

**Fig. 2:**
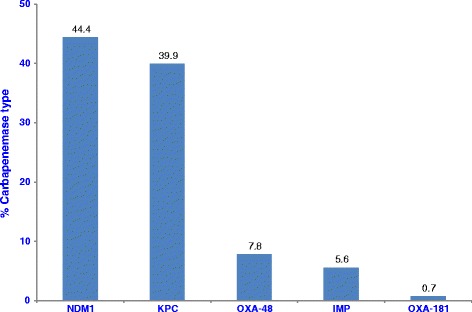
Carbapenemase types isolated

The distribution of isolation sites of the 268 isolates are shown in Fig. 1. CRE was commonly isolated from stool/rectal swab (61.6 %), urine (13.1 %) and other clinical sites (25.3 %). NDM-1 was the commonest CRE type seen (44.4 %), followed by KPC (39.9 %) whilst OXA-48 only constituted 7.8 % (Fig. 2).

#### Risk factor analysis

The mean total length of hospital stay is significantly longer in CRE cases than controls (48.0 vs 3.9 days, *p* < 0.001). Significant risk factors of the univariate analysis were summarized in Table 2. Higher proportions of CRE cases had an exposure to health care with history of previous overseas hospitalization (OR: 33.667; 95 % CI: 4.539–259.700), admission to ICU (OR: 11.899; 95 % CI: 4.986–28.399) and HD/ICA (OR: 6.557; 95 % CI: 4.057–10.596), with need for urinary catheter (OR: 4.239; 95 % CI: 2.630–6.831), drains (OR: 3.146; 95 % CI: 1.534–6.450) and enteral feeding (OR: 5.554; 95 % CI: 2.793–11.044). CRE cases were less likely to be ambulant upon arrival (OR: 0.337; 95 % CI: 0.221–0.513), and more likely to have comorbidities such as cardiovascular disease (OR: 1.868; 95 % CI: 1.256–2.778) and hematology conditions (OR: 4.328; 95 % CI: 1.592–11.769). They were also exposed to immunosuppressive treatments of DXT (OR: 2.230; 95 % CI: 1.057–4.706) and steroid (OR: 3.202; 95 % CI: 1.606–6.384). CRE cases were also exposed to a variety of antibiotics classes which included β-lactam/β-lactamase inhibitor combinations (OR: 3.676; 95 % CI: 2.432–5.557) and fluoroquinolones (OR: 2.018; 95 % CI: 1.111–3.663). In addition, CRE cases harbored other MDROs: MRSA (OR: 2.051; 95 % CI: 1.213–3.468) and VRE (OR: 7.411; 95 % CI: 2.825–19.441) and were exposed to invasive procedures of bronchoscopy (OR: 6.047; 95 % CI: 2.045–17.881).

**Table 2:** Univariate analysis of risk factors associated with CRE carriage

Risk factors	CP-CRE patients	Control	X = value/Mann-Whitney U	Odds Ratio
	(*N* = 203)	(*N* = 203)	(*p*-value/Fisher Exact)	[95 % Cl]
	n (%)	n (%)		
Total length of stay ± SD	48.0 ± 59.5	3.9 ± 3 8	U: 3223	1.266 [1.197–1.340]
(days)	(39.8–56.3)	(3.4–4.5)	(<0.001)	(<0.001)
(moan days 95 % confidence interval)				
History of previous overseas hospitalisation	29 (14.3 %)	1 (0.5 %)	28.218 (<0.001)**	33.667 [4.539–259.700]
History of travelling in past 90 days	29 (14.3 %)	15 7.4 %)	4.996 (0.025)*	2.089 [10.83.4.028]
Ambulant on arrival	103 (50.7 %)	153 (75.4 %)	26.432 (<0.001)**	0.337 [0.221–0.513]
Admission to CU	54 (26.6 %)	6 (3.0 %)	45.059 (<0.001)**	11.899 [4.986–28.399]
Mean ICU stay ± SD, day	9.6 ± 12.2	1.2 ± 1.0	*U*: 46.0 (<0.004)**	-
(mean days 95 % confidence interval)	(6.3—13.0)	(0.1—2.2)		
Admission to HD/ICA	106 (52.2 %)	29 (14.3 %)	65.797 (<0.001)**	6.557 [4.057–10.596]
Mean HD/ICA stay ± SD, day	5.3 ± 6.1	2.0 ± 1.7	9.0 (0.372)	-
(mean days 95 % confidence interval)	(4.1–6.5)	(1.4–2.7)		
Cardiovascular disease comorbidity	105 (51.7 %)	74 (36.5 %)	9.602 (0.002)**	1.868 [1.256–2.778]
Diabetes mellitus comorbidity	67 (33.0 %)	58 (28.6 %)	0.936 (0.333)	1.232 [0.807–1.870]
Malignancy cormorbidity	57 (28.1 %)	41 (20.2 %)	3.443 (0.064)	1.543 [0.974–2.442]
Immunodeficiency comorbidity	10 (4.9 %)	2 (1.0 %)	5.496 (0.019)*	5.207 [1.126–24.072]
Haematology comorbidity	20 (9.9 %)	5 (2.5 %)	9.591 (0.002)**	4.328 [1.592–11.769]
Exposure to DXT	23 (11.3 %)	11 (5.4 %)	4.622 (0.032)*	2.230 [1.057–4.706]
Exposure to steroid	34 (16.7 %)	12 (5.9 %)	11.866 (0.001)**	3.202 [1.606–6.384]
Exposure to chemotherapy	15 (7.4 %)	16 (7.9 %)	0.035 (0.852)	0.933 [0.448–1.941]
Exposure to penicillin antibiotics	33 (16 3 %)	7 (3.4 %)	18.747 (<0.001)**	5.435 [2.344–12.604]
Exposure to β-lactam/β-lactamase inhibitor combinations antibiotics	122 (60.1 %)	59 (29.1 %)	39.568 (<0.001)**	3.676 [2.432–5.557]
Exposure to cephalosporin antibiotics	65 (32.0 %)	43 (21.2 %)	6.106 (0.013)*	1.753 [1.120–2.742]
Exposure to carbapenems antibiotics	62 (30.5 %)	5 (2.5 %)	58.077 (<0.001)**	17.413 [6.826–44.417]
Exposure to fluoroquinolones antibiotics	35 (17.2 %)	19 (9.4 %)	5.468 (0.019)*	2.018 [1.111–3.663]
Exposure to glycopeptide antibiotics	69 (34.0 %)	4 (2.0 %)	70.564 (<0001)**	25.618 [9.132–71.865]
Exposure to metronidazole antibiotics	36 (17.7 %)	15 (7.4 %)	9.889 (0.002)**	2.702 [1.428–5.110]
Exposure to aminoglycosides antibiotics	17 (8.4 %)	1 (0.5 %)	14.882 (<0.001)**	18.462 [2433–140,097]
Presence of central line device	80 (39.4 %)	11 (5.4 %)	67.433 (<0.001)**	11.353 [5.811–22.179]
Presence of urinary catheter device	86 (42.4 %)	30 (14.8 %)	37.842 (<0.001)**	4.239 [2.630–6.831]
Presence of ETT device	42 (20.7 %)	6 (3.0 %)	30.620 (<0.001)**	8.565 [3.55 1–20.658]
Presence of intra-arterial line device	24 (11.8 %)	5 (2.5 %)	13.406 (<0.001)**	5.309 [1.984–14.2 11]
Presence of drains device	31 (15.3 %)	11 (5.4 %)	10.623 (0.001)**	3.146 [1.534–6.450]
Presence of enteral feeding device	49 (24.1 %)	11 (5.4 %)	28.240 (<0.001)**	5.554 [2.793–11.044]
Presence of additional MRSA	47 (23.2 %)	26 (12.8 %)	7.365 (0.007)**	2.051 [1.213–3.468]
Presence of additional VRE	32 (15.8 %)	5 (2.5 %)	21.678 (<0.001)**	7.411 [2.825-19.441]
Exposure to bronchoscopy procedure	22 (10.8 %)	4 (2.0 %)	13.314 (<0.001)**	6.047 [2.045–17.881]
Exposure to gastroscopy procedure	18 (8.9 %)	10 (4.9 %)	2.455 (0.117)	1.878 [0.845–4.175]
Exposure to colonoscopy procedure	4 (2.0 %)	6 (3.0 %)	0.410 (0.522)	0.660 [0.183–2.375]
Exposure to angiogram procedure	9 (4.4 %)	6 (3.0 %)	0.623 (0.430)	1.523 [0.532–4361]
Underwent surgery	141 (69.5 %)	146 (71.9 %)	0.297 (0.586)	1.126 [0.734–1.727]

*p < 0.05 (Significance of association; 2-tailed), **p < 0.01 (Significance of association; 2-tailed)

The results for the multivariate analysis were presented in Table 3. Upon adjustment for total length of hospital stay, exposure to antibiotics penicillin (OR: 4.640; 95 % CI: 1.529–14.079] and glycopeptide (OR: 5.162; 95 % CI: 1.377–19.346) and presence of central line device (OR: 3.117; 95 % CI: 1.167–8.330) emerged as significant independent predictors associated with CRE.

**Table 3:** Multivariate analysis of risk factors

Risk factor (Multivariable model)	Adjusted Odds Ratio
	[95 % Confidence Interval]
	(Sig)
Exposure to antibiotics penicillin	4.640 [1.529–14.079] (0.007)*
Exposure to antibiotics glycopeptides	5.162 [1.377–19.346] (0.015)**
Presence of central line device	3.117 [1.167–8.330] (0.023)**

*p < 0.01; **p < 0.05 (Significance of association; 2-tailed)

#### Outcomes

There were 37 (18.2 %) mortalities amongst the CRE cases. CRE cases had ~3.5 times odds of fatality adjusted for length of hospital stay (adjusted OR: 3.532; 95 % CI: 1.281–9.741; *p*: 0.015) compared to controls. No outbreak was noted during the study period.

## Discussion

Interestingly, the results of our case-control study revealed different risk factors from other investigators. Twenty-nine of our 203 cases (85.7 %) had no history of overseas travel in the past 90 days before admission and had no previous overseas hospitalization. Hence, it is likely that CRE is already in our community setting.

Our study identified similar risk factors that other investigators have highlighted-exposure to antimicrobials, especially carbapenems and fluoroquinolones [12–15], admission to ICU [16]; presence of indwelling devices [17] e.g. central line, urinary catheter, endotracheal tube and the enteral feeding tube. In addition, our study identified unique risk factors viz. hematology patients and those with immunodeficiency. This may be explained by the recurring admissions and discharges as well as a relatively longer length of stay in this patient population exposing them to greater risk than the general patient population.

Our multivariate analysis showed similar findings with other investigators [18, 19] that antimicrobial usage, increased risk for CRE colonization. Penicillin use was associated with increased risk for CRE colonization among patients admitted to the hospital, as in our study. From 2011 to 2013, there were 11 patients with CP-CRE bacteraemia; four of these (36.3 %) had a central venous pressure (CVP) line. Indwelling devices are recognised risk factors for healthcare-associated infections [20, 21]. Hence, it is not surprising to see the presence of a CVP line as an independent risk factor for CRE infection. This highlights the importance of safe patient care practices especially in the care of devices as well as the significance of an antimicrobial stewardship program in the strategy for the prevention of CRE infections.

We conducted a preliminary data analysis for risk factors from the 2011–2012 data and identified criteria for active surveillance for CRE as part of an effort to reduce the incidence of CRE. From January 2013, the criteria used were history of overseas travel in the past one year, transfers from overseas hospital and history of admissions to private hospitals in Singapore in past one year. This was modified to include admissions to ICU, high dependency units and intermediate care units (October 2013); renal (March 2014), haematology (April 2014), oncology (June 2014). Since then, our healthcare-associated CRE has stabilized to 0.28 per 1000 patient days (2014) from previous rates of 0.03 and 0.26 respectively (2012, 2013). We were able to institute prompt isolation and control measures upon knowing the active surveillance screening results. This helped to prevent horizontal transmission between patients in our general wards, where most patients are housed in open wards with 4–8 beds in a cubicle.

Our study has several limitations. Firstly, CRE was identified from clinical specimens submitted to the Microbiology Laboratory. Active surveillance for CRE carriage was not done during 2011–2012 and the CRE carriage rate could have been higher than found. Secondly, because active surveillance for CRE was not done at admission, we were not able to determine if the patients had acquired it during their inpatient stay. A case control study involving multiple centers or over a longer period may help to overcome the problem of small sample size. Our study is underpowered at 47.4 % (α: 0.05) as we only had 203 patients in each arm. For power to be at 80 %, we would need 855 patients in each arm [22]. This study investigated the risk factors for being colonized with CRE in our patient population. Acquisition was not studied as exit swabs were not done for those who had entry screening swabs done. Hence, it will not be possible to correlate the link between the risk factors and acquisition.

## Conclusions

The global increase in CRE in many healthcare facilities poses challenges to infection control and infectious disease professionals. Risk-based screening is one strategy that has been used to limit the spread of CRE in healthcare settings [18, 19, 23]. We have demonstrated the value of understanding local epidemiology to help modify our risk-based screening as a strategy to limit the spread of CRE. Hence, we recommend targeted screening strategies to identify patients colonized with CRE at the time of admission to a healthcare institution as a component of a CRE reduction program.

## References

[1] Wei ZQ, Du XX, Yu YS (2007). Plasmid-mediated KPC-2 in a *Klebsiella pneumoniae* isolate from China. Antimicrob Agents Chemother.

[2] Rhee JY, Park YK, Shin JY (2010). KPC-producing extreme drug-resistant *Klebsiella pneumoniae* isolate from a patient with diabetes mellitus and chronic renal failure on hemodialysis in South Korea. Antimicrob Agents Chemother.

[3] Chia JH, Su LH, Lee MH (2010). Development of high-level carbapenem resistance in *Klebsiella pneumoniae* among patients with prolonged hospitalization and carbapenem exposure. Microb Drug Resist.

[4] Balm MN, Ngan G, Jureen R, Lin RT, Teo J (2012). Molecular characterization of newly emerged blaKPC-2-producing *Klebsiella pneumoniae* in Singapore. J Clin Microbiol.

[5] Yong D, Toleman MA, Giske CG (2009). Characterisation of a new metallo- β -lactamase gene, bla NDM-1, and a novel erythromycin esterase gene carried on a unique genetic structure in *Klebsiella pneumoniae* sequence type 14 from India. Antimicrob Agents Chemother.

[6] Chien JM, Koh TH, Chan KS (2012). Successful treatment of NDM-1 *Klebsiella pneumoniae* bacteraemia in a neutropenic patient. Scand J Infect Dis.

[7] Ng E, Earnest A, Lye DC (2012). The excess financial burden of multidrug resistance in severe gram-negative infections in Singaporean hospitals. Ann Acad Med Singapore.

[8] Ben-David D, Maor Y, Keller N (2010). Potential role of active surveillance in the control of a hospital-wide outbreak of carbapenem-resistant *Klebsiella pneumoniae* infection. Infect Control Hosp Epidemiol.

[9] Munoz-Price LS, Cuesta CDL, Adams S (2010). Successful eradication of a monoclonal strain of *Klebsiella pneumoniae* during a *K. pneumoniae* carbapenemase-producing *K. pneumoniae* outbreak in a surgical intensive care unit in Miami, Florida. Infect Control Hosp Epidemiol.

[10] Lerner A, Abu-Hanna K, Meitus I (2013). Environmental contamination by carbapenem-resistant *Enterobacteriaceae*. J Clin Microbiol.

[11] Kotsanas D, Wijesooriay WR, Korman TM (2013). “Down the drain”: carbapenem-resistant bacteria in intensive care unit patients and handwashing sinks. Med J Aust.

[12] Kritsotakis EI, Tsioutis C, Roumbelaki M (2011). Antibiotic use and the risk of carbapenem-resistant extended spectrum-β-lactamase-producing *Klebsiella pneumoniae* infection in hospitalized patients: results of a double case–control study. J Antimicrob Chemother.

[13] Schechner V, Kotlovsky T, Tarabeia J (2011). Predictors of rectal carriage of carbapenem-resistant *Enterobacteriaceae* (CRE) among patients with known CRE carriage at their next hospital encounter. Infect Control Hosp Epidemiol.

[14] Patel N, Harrington S, Dihmess A (2011). Clinical epidemiology of carbapenem-intermediate or –resistant *Enterobacteriaceae*. J Antimicrob Chemother.

[15] Liu S, Chang H, Chia J (2012). Outcomes and characteristics of ertapenem non-susceptible *Klebsiella pneumoniae* bacteremia at a university hospital in Northern Taiwan: a matched case-control study. J Microbiol Immunol Infect.

[16] Lee GC, Lawson KA, Burgess DS (2013). Clinical epidemiology of carbapenem-resistant *Enterobacteriaceae* in community hospitals: a case-case-control study. Ann Pharmacother.

[17] Bhargava A, Hayakawa K, Silverman E (2014). Risk factors for colonization due to carbapenem-resistant *Enterobacteriaceae* among patients exposed to long-term acute care and acute care facilities. Infect Control Hosp Epidemiol.

[18] Borer A, Saidel-Odes L, Eskira S (2012). Risk factors for developing clinical infection with carbapenem-resistant *Klebsiella pneumonia*e in hospital patients initially only colonized with carbapenem-resistant *K pneumoniae*. Am J Infect Control.

[19] Falagas ME, Rafailidis PI, Kofteridis D (2007). Risk factors of carbapenem-resistant *Klebsiella pneumoniae* infections: a matched case control study. J Antimicrob Chemother.

[20] Rosenthal VD, Maki DG, Salomao R (2006). Device-associated nosocomial infections in 55 intensive care units of 8 developing countries. Ann Intern Med.

[21] Moreno CA, Rosenthal VD, Olarte N (2006). Device-associated infection rate and mortality in intensive care units of 9 Colombian hospitals: findings of the International Nosocomial Infection Control Consortium. Infect Control Hosp Epidemiol.

[22] Whitley E, Ball J (2002). Statistics Review 4. Sample size calculations. Crit Care.

[23] Chitnis AS, Caruthers PS, Rao AK (2012). Outbreak of carbapenem-resistant *Enterobacteriaceae* at a long-term acute care hospital: sustained reductions in transmission through active surveillance and targeted interventions. Infect Control Hosp Epidemiol.

